# Light-Assisted Enhancement of Gas Sensing Property for Micro-Nanostructure Electronic Device: A Mini Review

**DOI:** 10.3389/fchem.2021.811074

**Published:** 2021-12-24

**Authors:** Zongtao Ma, Ziying Wang, Lingxiao Gao

**Affiliations:** ^1^ State Key Laboratory of Reliability and Intelligence Electrical Equipment, Hebei University of Technology, Tianjin, China; ^2^ National Engineering Research Center for Technological Innovation Method and Tool, School of Mechanical Engineering, Hebei University of Technology, Tianjin, China; ^3^ School of Electronics and Information Engineering, Hebei University of Technology, Tianjin, China

**Keywords:** light irradiation, micro-nanostructure, gas sensing devices, sensor, metal oxide semiconductor

## Abstract

In recent years, gas sensing electronic devices have always attracted wide attention in the field of environment, industry, aviation and others. In order to improve the gas sensing properties, many micro- and nano-fabrication technologies have been proposed and investigated to develop high-performance gas sensing devices. It is worth noting that light irradiation is an effective strategy to enhance gas sensitivity, shorten the response and recovery time, reduce operating temperature. In this review, firstly, the latest research advances of gas sensors based on different micro-nanostructure materials under UV light and visible light activation is introduced. Then, the gas sensing mechanism of light-assisted gas sensor is discussed in detail. Finally, this review describes the present application of gas sensors with improved properties under light activation assisted conditions and the perspective of their applications.

## Introduction

With the rapid improvement of economy and the quality of human life, people have realized that environmental pollution has caused irreparable damage to the Earth. Harmful gases from coal-fired power stations, garbage incineration, automobile exhaust and industrial waste gas not only pose a threat to the environment but also endanger human health (Cheng et al., 2021a). Therefore, the development of harmful gas detection technology is of great significance.

The metal oxide semiconductor (MOS) sensors have many advantages of small size, high response, fast response and so on, which attract great interest in real-time detection of different gases. However, the high operating temperature can reduce the life of the device and sensitivity. Consequently, researchers began to explore gas sensing devices by putting forward a large number of theoretical methods and experimental schemes, which can work at low temperature or even room temperature (RT). In recent years, with the continuous development of nanotechnology and nanomaterials, the research of RT gas sensor has also got significant progress include morphological control (Wang et al., 2021, noble metal surface modification (Wang et al., 2019a) or doping and the formation of heterostructures (Wang et al., 2019b). Furthermore, light activation is an effective method to improve the performance of MOS sensors. The optical irradiation of MOS sensor can change the surface electronic properties by adjusting the concentration of optical carriers in MOS, so as to promote the interaction between molecule and sensor layer (Kumar et al., 2020). Herein, we will summarize the latest progress of photoactivated RT MOS sensors in the past few years.

Recently, many reports have confirmed that ultraviolet (UV) irradiation can indeed improve the performance of MOS sensors, including higher sensitivity, shorter recovery time and lower power consumption. UV excitation can increase the density of free electron-hole pairs and lead to photodissociation of the target gas and chemical surface adsorbents (Zhai et al., 2018; Park et al., 2014; Hyodo et al., 2017). However, the harmful effects of UV light on human skin and eyes remain an acute problem, and UV light accounts for only 5–7% of the total energy from sunlight. Therefore, additional UV-LED light sources are required, which presents a new challenge to the size design of the sensor. By contrast, visible light is superior to UV light in terms of energy acquisition and energy utilization. More importantly, it does no harm to human health. Similarly, visible light activation of narrow band gap metal oxides for RT gas sensing has been explored by numerous researchers in the past few years (Hasani et al., 2015; Li et al., 2018; Song et al., 2020). While visible light activation is an effective way to improve the performance of MOS sensors, it still faces great challenges to widely apply it in real environment.

In this paper, we discuss RT gas sensors under light assisted conditions. This review is divided into three parts: firstly, we focus on the latest progress of gas sensors based on MOS, noble metal doped MOS and MOS with heterojunction structure at room temperature by different wavelength light sources (UV light and visible light). Secondly, we describe the gas sensing mechanism under light-assisted condition. Finally, we introduce the application status and future prospects of gas sensors under light-assisted conditions.

## Light Activation Gas Sensors Based on Different Micro-Nanostructure Materials

Many studies have shown that light irradiation is one of the effective ways to improve the gas sensitivity of pure MOS, noble metal doping MOS nanostructure and MOS with heterostructure can be improved under the condition of light activation, which will be described one by one below.

### Gas Sensors Based on Pure Metal Oxide Nanostructure

**FIGURE 1:**
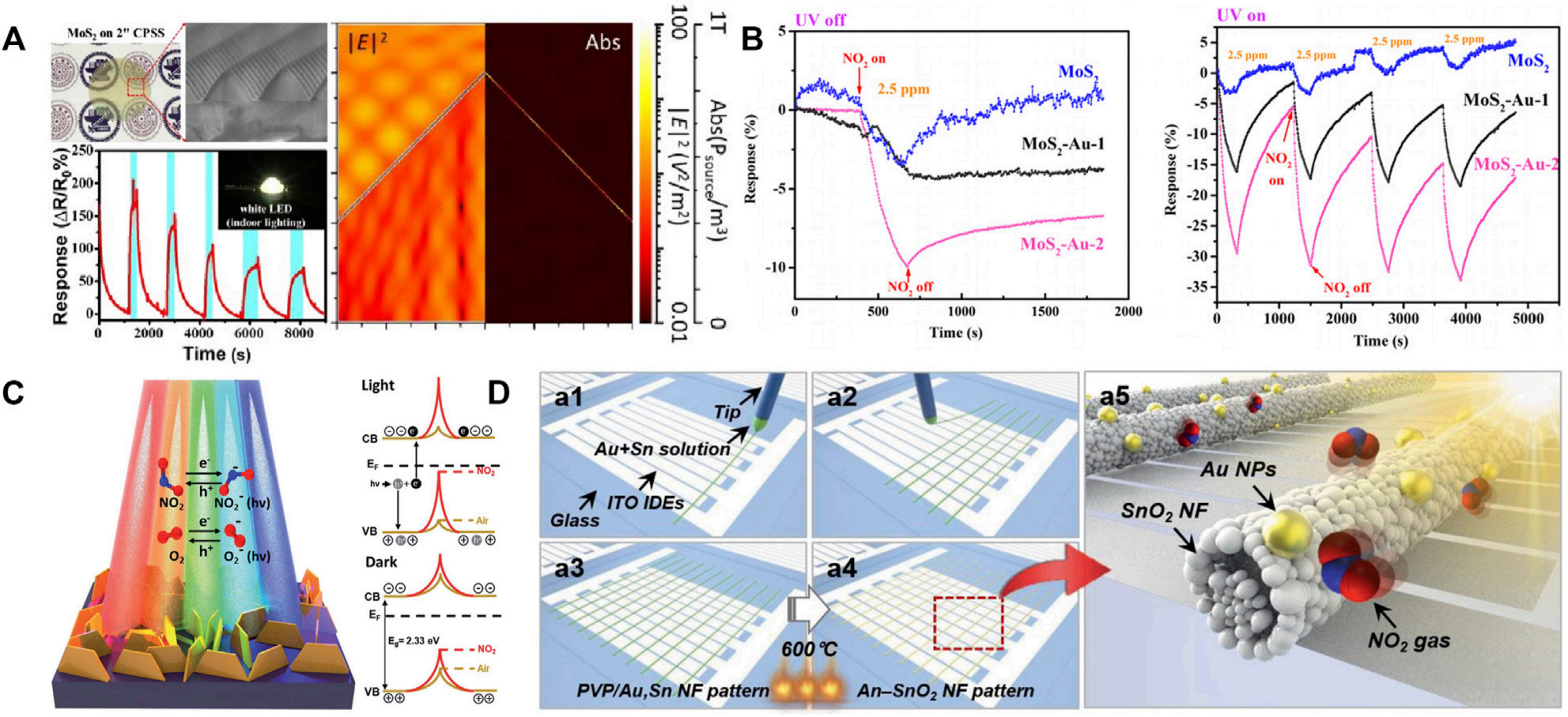
**(A)** Microscopic characterization of MoS_2_ and gas sensing performance under white light, Simulation results for the Flat-MoS_2_ and Cone-Shaped-MoS_2_ gas sensors by finite difference time domain (FDTD) illuminated by the light with the wavelength of 550 nm. Reproduced with permission from ref. 13. Copyright 2019, Royal Society of Chemistry. **(B)** Schematic illustrations of edge-enriched SnS_2_ NFs for visible light activated NO_2_ gas sensor. Reproduced with permission from ref. 14. Copyright 2021, Royal Society of Chemistry. **(C)** Sensing performance of MoS_2_ and MoS_2_-Au sensors toward NO_2_ gas with no UV illumination. Reproduced with permission from ref. 17. Copyright 2018, American Institute of Physics. **(D)** Schematic illustration for (left) the experimental procedures to fabricate nanofiber pattern (NF-P) sensors via near-field electrospinning (NFES) and (right) NO_2_ detection of nanofiber pattern (NF-P) sensors under visible light illumination. Reproduced with permission from ref. 18. Copyright 2021, WILEY-VCH.

In 2008, Costello and his colleagues firstly confirmed that UV light can improve the RT sensitivity of ZnO sensor. The ZnO sensor could detect 1 ppb of acetaldehyde and acetone and obtained adjustable sensitivity though manipulating the intensity of UV light (De Lacy Costello et al., 2008). Chen et al. demonstrated the gas sensor based 3D cone-shaped MoS_2_ bilayer showed high response (∼470%) and short response time (∼25 s) after exposure to 1 ppm of NO under UV light (Chen et al., 2019). As shown in Figure 1A, it is explained that the excellent NO sensing properties is due to the three-dimensional light scattering effect attracted by UV light, which further enhances the light absorption.

Because of the health hazards and low utilization of UV light, researchers began to explore the replacement of UV light with visible light. Li et al. prepared a highly ordered CdS nanoflakes array by using Chemical Vapor Deposition (CVD) technology and studied its gas sensing characteristics (Li et al., 2018). It is found that the sensor has good working potential under natural solar lamps and can be used for outdoor environment monitoring. The excellent gas performances are attributed to the low band gap energy (2.4 eV) and the unique morphology of CdS. These inherent properties of CdS can enhance light absorption and conductivity. Wang et al. successfully prepared SnS_2_ nanoflowers by solvothermal synthesis (Eom et al., 2021). In Figure 1B, it is confirmed that the high absorbance of SnS_2_ in the visible region triggered the generation of carriers, which could decrease the resistance and enhance the gas sensing characteristics. Friedman et al. fabricated MoS_2_ by mechanical stripping and tested the sensitivity of the sensor under visible light (Friedman et al., 2014). They observed a 10-fold difference in the sensitivity of the sensors to trimethylamine before and after light exposure. The improved gas sensitivity of MoS_2_ sensors comes from photoexcitation.

### Gas Sensors Based on Noble Metal Doping Nanostructure

Many studies have demonstrated that the modification of noble metal and light irradiation have synergistic effect on improving the gas sensing performance of pure MOS. On one hand, Li et al. fabricated a sensor based on Au/ZnO porous octahedron (POHs). Compared with the sensor based pure ZnO POH, the response of the sensor based on Au/ZnO POHs to formaldehyde is significantly improved under UV light (Tsai et al., 2018). Zhou et al. reported a UV-assisted, recoverable, highly sensitive and selective NO_2_ gas sensor based on Au-MoS_2_ nanocomposite (Zhou et al., 2018a). In Figure 1C, the Au-MoS_2_ sensor has a three-fold enhanced response to NO_2_, full recovery property and good repeatability under UV light. The detection of NO_2_ under UV light provides an alternative strategy for the design of single multifunctional optoelectronic devices.

On the other hand, Kim et al. reported the detection of NO_2_ gas by a one-dimensional Au-SnO_2_ nanofiber sensor under visible light at RT (Lim et al., 2021). The sensor shows a high degree of selectivity, sensitivity and repeatability in response to NO_2_ at sub ppm levels. As shown in Figure 1D, the excellent RT NO_2_ properties are in connection with the effects of Au nanoparticles from local surface plasmon resonance (LSPR) in visible light. This work makes the new transparent design possible for oxide gas sensors without external heaters or light sources. Additionally, Chen et al. developed a high-performance visible light activated NO_2_ gas sensor based on LSPR and increased surface oxygen vacancy (Chen et al., 2020). The results show that Au NPs modification can significantly improve the visible photosensitivity of ZnO films compared with pure ZnO films. It provides an effective way to construct high-performance photoactivated gas sensor.

### Gas Sensors Based on Heterojunction Nanostructure Composite

Both the construction of heterostructure and light irradiation can increase active sites and charge transfer of pure metal oxide semiconductor. The heterostructure of MOS can enhance charge transduction and adjust grain boundary potential barrier, which is beneficial to improve the gas sensitivity. For one thing, Chang et al. prepared hollow ZnO/MoS_2_ nanosheets with core/shell heterostructure (Chang et al., 2020). The high acetone response and fast response/recovery rate are caused by the fast gas transport channel and the n-p heterojunction of MoS_2_ nanosheets. Moreover, UV light is introduced to further improve the acetone reaction and greatly reduce the operating temperature. The optical diffraction and reflection caused by the decoration of layered MoS_2_ nanosheets can significantly improve the light capture. Yang et al. successfully synthesized hollow ZnO-SnO_2_ heterostructure for triethylamine (TEA) (Yang et al., 2018). Particularly, the sensor based ZnO-SnO_2_ showed short response time (1.8 s) and recovery time (18 s) under UV light.

For another, Liu et al. reported hollow SnO_2_@SnS_2_ nanostructures prepared by one-step hydrothermal method (Liu et al., 2020). The sensor based on SnO_2_@SnS_2_ has a fast response rate and good recovery ability to ppb-level NO_2_ under visible light at RT. The excellent sensing performance of SnO_2_@SnS_2_ sensor is due to the hollow and porous heterojunction structure, which is conducive to gas diffusion, especially visible light assisted to promote charge transfer and gas desorption. Chen et al. prepared gas-sensitive ZnO nanorods with sea urchin-level mesoporous structure modified by PbS quantum dots (QDs) (Chen et al., 2018). The PbS QDs have a narrow band gap shown in the Near Infrared Spectroscopy Analysis (NIR) spectrum. Compared with the original ZnO nanorods, the sensor based ZnO/PbS nanocomposites have a higher response and faster response/recovery speed to ppm level NO_2_ under visible light.

## Sensing Mechanism of Light-Assisted Gas Sensor

Light activation has been proved to be an effective way to improve the performance of gas sensors, but the relationship between the types of light source, the structure of materials and the improvement of gas sensing properties are not clear. The explanations for the improved gas sensitivity of UV light are as follows:

Firstly, Gong et al. proposed the gas sensor based on a novel ZnO hybrid with nanowire/optical fiber (Gong et al., 2017). The results show that UV irradiation can improve the sensitivity and shorten the response time, and the sensor also has good long-term stability. UV irradiation can respond to gas with ppb-level concentration at low temperature. The electrons in the ZnO hybrid excited by UV irradiation promote the reduction of ethanol gas, leading to the higher gas performance. Therefore, the irradiation of UV will generate more conductive electrons, improve the conductivity of the sensing materials and promote the electron transfer under UV irradiation, thus improving the response of the gas sensor. Secondly, Zhou et al. have proposed that the increased sensitivity is ascribed to the increase in the number of active adsorption sites and the introduction of active catalysts such as Au, Ag, Pd and other noble metals (Zhou et al., 2018a). For example, Au nanoparticles can accelerate the capture of more photons, resulting in additional photoexcited carriers to promote the interaction between gas and sensing materials. The efficient separation of photoexcited carriers at the interface of MoS_2_/Au also contributes to rapid and complete recovery under UV light. Finally, Zhou et al. explained that UV light can excite electron-hole pairs in ZnO (n-type) and MoS_2_ (p-type) (Zhou et al., 2018b). The photoexcited electrons in the conduction band can be easily transferred to ZnO by the built-in field, which is similar to the excited hole in the valence band of ZnO to the excited hole of MoS_2_. Therefore, light carriers can be effectively separated at the interface of heterojunction in order to improve the gas sensing properties.

The explanations for the improved gas sensitivity of visible light are as follows: Primarily, the explanations given by Li et al. are as follows: The photon energy of visible light greater than the band gap of semiconductor can produce electron-hole pairs. Electrons and holes cannot recombine efficiently, and the electrons of light source move to the conduction band and then are accumulated in the conduction band. This results in the high concentration of electrons in the conduction band and the high conductivity of the semiconductor. Therefore, the sensitivity of the gas sensor is significantly increased under visible light (Li et al., 2018). Posteriorly, Chen et al. developed a high-performance visible light activated NO_2_ sensor based on LSPR and increased surface oxygen vacancy (Chen et al., 2020). The mechanism of visible light activated by LSPR absorption are illustrated. At the interface of metal and semiconductor, the Fermi level of ZnO is transferred to a positive charge when electrons transfer from ZnO nanorods to Au nanorods, resulting in an upward-bending band interface and a Schottky barrier. The photoexcited electrons generated in Au can overcome the blocking and injection of conduction band in ZnO, making more free electrons can participate in the chemical reaction with the surface adsorbed O_2_ and NO_2_ molecules. Niu et al. prepared vertically stacked MoS_2_/GaSe heterojunction by all-dry transfer method (Niu et al., 2021). When the p-n junction between MoS_2_ and GaSe is exposed to visible light, electron-hole pairs in both MoS_2_ and GaSe layers are generated by light and tend to migrate at the interface. Due to the built-in electric field, electrons transfer to n-type MoS_2_ and holes transfer to p-type GaSe, resulting in carrier separation. NO_2_ has a strong electron affinity and can easily capture electrons from the conduction band of the material. In general, the separation of the light source carrier and the built-in electric field acts as a driving force to drive the gas sensing behavior.

The possible mechanism and reasons for the superior gas performance of light irradiation can be explained as follows:

1) For pure metal oxide nanostructure, the gas sensing performance depends on the amount of oxygen active substance adsorbed on the nanostructure surface. Under dark conditions, the concentration of free carriers inherent in the nanostructure is low, resulting in less utilization of the active site to produce absorbed oxygen. In contrast, with the increase of the number of free carriers under light irradiation, the surface absorption site improves the chemical activity of the surface and thus enhances the gas sensitivity.
2) For noble metal doping nanostructure, the LSPR effect not only enhances the absorption of light, but also inhibits the reorganization of electron-hole pairs produced by light. The intermediate-thermal electrons generated by LSPR absorption can overcome the Schottky barrier at the noble metal/oxide junction and inject into the oxide conduction band. Therefore, more surface adsorbed oxygen is formed on the oxide surface, leading to a stronger sensing reaction.
3) For heterojunction nanostructure composite, the formation of heterojunction can promote the separation of electron-hole pairs, effectively accelerate the electron conversion between different particles, and improve the response rate. When the heterojunction is exposed to light irradiation, electron-hole pairs in the semiconductor are generated by light and tend to migrate at the interface. Due to the built-in electric field, the electron transfer rate is enhanced, resulting in carrier separation and improving the gas sensitivity.

## The Present Application of Gas Sensors Under Light Activation

As essential for environmental monitoring, process control and early diagnosis of human disease, gas sensors under light activation are used to sense volatile organic compounds (VOCs). Wu et al. demonstrated a specific and highly sensitive detection of ketones using two-dimensional (2D) MoTe_2_ (Wu et al., 2018). They also investigated the effect of UV activation on the sensing performance of VOCs. Activation of UV light results in high sensitivity, low detection limit (∼0.2 ppm) to acetone and high stability even in high humidity, which is crucial for early diagnosis of diabetes. Zhao et al. demonstrated a flexible, transparent and high-performance gas sensor based In_2_Se_3_ using a simple pulsed laser deposition (PLD) method (Zheng et al., 2017). The gas sensor can work under the activation of visible light and shows excellent performance in detecting acetylene gas. No significant degradation of sensing performance was observed even after 100 bending cycles. The In_2_Se_3_ sensor has an average transmittance of 64% in the visible light range (400–800 nm). In addition, they found that the sensitivity, response and recovery rate of the sensor based In_2_Se_3_ depend on the light intensity. The excellent performance of In_2_Se_3_ sensors on flexible substrates provides a brilliant future for practical applications in wearable optoelectronic systems. Cheng et al. also prepared CuO flower-like materials (FMs) by hydrothermal method, and then obtained Au@CuO FMs by *in-situ* reduction reaction (Cheng et al., 2021b). The performance of the sensor based Au@CuO FMs was evaluated by detecting volatile and toxic gases such as ethanol, isopropanol, methanol and formaldehyde under UV light. It was found that the sensor based Au@CuO FMs had a higher response (95.3) than the sensor based CuO FMs at a concentration of 1,000 ppm, while the sensor based Au@CuO FMs had a response of 174 under UV light. Therefore, Au@CuO FMs is a promising ethanol detection method. In summary, light irradiation as an effective means enables gas sensors to maintain better performance in environmental monitoring and human health protection.

## Conclusion

In this paper, we first introduce the latest research progress of gas sensors with different microstructures (pure metal oxide nanostructure, noble metal doping nanostructure, heterojunction composite nanostructure) activated by UV and visible light. In the next place, we introduce the light activation mechanism of gas sensors with different structures from the perspective of microscopic mechanism. Finally, several applications of photoactivated gas sensors in environmental monitoring, process control and early diagnosis of human diseases are listed.
